# AI-Enhanced Dyscalculia Screening: A Survey of Methods and Applications for Children

**DOI:** 10.3390/diagnostics14131441

**Published:** 2024-07-05

**Authors:** Shashi Bhushan, Sharmila Arunkumar, Taiseer Abdalla Elfadil Eisa, Maged Nasser, Anuj Kumar Singh, Pramod Kumar

**Affiliations:** 1 Department of Computer & Information Sciences, Universiti Teknologi Petronas, Seri Iskandar 32610, Perak, Malaysia; maged.nasser@utp.edu.my; 2 Raj Kumar Goel Institute of Technology, Ghaziabad 201017, Uttar Pradesh, India; sharmila1ece@gmail.com; 3 Department of Information Systems—Girls Section, King Khalid University, Mahayil 62529, Saudi Arabia; teisa@kku.edu.sa; 4 School of Computing Science and Engineering, Galgotias University, Greater Noida 201310, Uttar Pradesh, India; mail2anujji@gmail.com; 5 Himalayan School of Science and Technology, Swami Rama Himalayan University, Dehradun 248016, Uttarakhand, India; tulasacademic@gmail.com

**Keywords:** AI (artificial intelligence), dyscalculia, dyslexia, specific learning disabilities, neurodevelopmental disorders, learning disabilities, early intervention, cognitive abilities, dysgraphia, mathematical comprehension

## Abstract

New forms of interaction made possible by developments in special educational technologies can now help students with dyscalculia. Artificial intelligence (AI) has emerged as a promising tool in recent decades, particularly between 2001 and 2010, offering avenues to enhance the quality of education for individuals with dyscalculia. Therefore, the implementation of AI becomes crucial in addressing the needs of students with dyscalculia. Content analysis techniques were used to examine the literature covering the influence of AI on dyscalculia and its potential to assist instructors in promoting education for individuals with dyscalculia. The study sought to create a foundation for a more inclusive dyscalculia education in the future through in-depth studies. AI integration has had a big impact on educational institutions as well as people who struggle with dyscalculia. This paper highlights the importance of AI in improving the educational outcomes of students affected by dyscalculia.

## 1. Introduction

Learning disabilities can significantly impact a person’s cognitive abilities, making it challenging to perform everyday tasks smoothly. Among the various types of learning disabilities, some of the most common ones include dyslexia, dysgraphia, and dyscalculia. Dyslexia is known as a reading disability and entails challenges in reading and processing sounds. Individuals with dyslexia may struggle to relate sounds to letters. Dysgraphia, often referred to as a writing disability, involves difficulties in writing and confusion between similar letters like “b” and “d”. Dyscalculia, another learning disability, impedes the grasp of mathematical concepts, making it hard for individuals to understand them. Dyscalculia is a specific kind of learning problem that affects how well a person understands and manipulates numbers and mathematical concepts. Basic arithmetic procedures, number sense, comprehending mathematical symbols and concepts, and problem-solving in mathematics can all be difficult for those with dyscalculia. This learning challenge is far more closely linked to how the brain processes numerical data than to intelligence. People who suffer from dyscalculia may find it difficult to determine time, count, comprehend mathematical symbols, and apply addition, subtraction, multiplication, and division to mathematical problems. They may also find it challenging to understand concepts related to measurement, estimation, and mathematical reasoning. Dyscalculia can vary in severity among individuals, and it can persist into adulthood if not properly addressed and accommodated. Early detection and assistance can assist people with dyscalculia in learning coping mechanisms and enhancing their mathematical abilities. Individualized learning strategies, multisensory teaching techniques, assistive technology, and specialized instruction can all help people with dyscalculia overcome obstacles and excel in mathematics and other related subjects. Dyscalculia affects about 6% of people with learning disabilities. As kids grow up, their brains get better at understanding numbers and math. Scientists have found that different parts of the brain work together when we learn and use math skills. To get good at math, our brain needs to develop different abilities. Paying attention helps us focus on math problems, while understanding language helps us understand what the math problems mean. Remembering numbers and doing math in our head (like adding or subtracting) is easier when our memory is strong. Seeing shapes and patterns helps us understand math concepts better, and using our fingers to count can also help us learn numbers early on. All of these skills improve as we grow older, and they play a big role in how well we understand and use math. For people with dyscalculia, these skills might not develop as quickly or as well, which can make math harder for them. That is why it is important to find ways to help them learn math in a way that works for them. Unlike dyslexia and dysgraphia detection, which do not typically involve complex scoring methods like dyscalculia, dyscalculia detection often employs various tests. The goal of this approach is to make the process of detection and assessment for medical professionals more efficient. The Woodcock–Johnson IV test is administered at the B.Y.L. Nair Ch. Hospital. This test determines if an individual has dyscalculia by having them complete a series of exams that get harder and harder. To diagnose dyscalculia in a patient, doctors had to manually compute test results under the previous system. This manual scoring process was tedious and prone to errors, potentially leading to incorrect evaluations. Patients experienced long waits for evaluation reports, as data were stored physically in hospital files. The initial project version used test results to calculate the probability of dyscalculia, eliminating the risk of manual errors and providing instant results to patients. To achieve more accurate findings, the dataset size has been increased in the current edition. The data kept in the model are encrypted, and the web application is password-protected to enhance privacy and patient data confidentiality. After examining the results of the Woodcock–Johnson Test, which is monitored by physicians to identify learning problems, curriculum-based tests (CBTs) might be necessary. Despite ongoing research, dyscalculia detection remains manual and time-consuming. Machine learning aids in assessing the significance of all tests for dyscalculia detection.

The organization of the paper unfolds as follows: In Section 2, there is an exploration of different kinds of mental disorders. Section 3 delves into the challenges of prevalence studies. In Section 4, the focus is on identifying the characteristics, causes, and therapeutic approaches of dyscalculia. Section 5 and Section 6 provide a treatment of dyscalulia and summarize the role of technologies in special education. Section 7, Section 8, Section 9, Section 10, Section 11 and Section 12 discuss the test to identify dyscalculia followed by the drawbacks of the existing system, literature survey, the use of AI in dyscalculia, dyscalculia screening and a discussion on the key discoveries and study outcomes. Finally, Section 13 wraps up with the conclusion, summaries, and areas for future work.

## 2. Different Kinds of Mental Disorders

A wide range of illnesses are classified as children’s and teenage mental disorders in the Diagnostic and Statistical Manual of Mental Disorders (DSM). Neurodevelopmental disorders such as dyslexia, autism, ADHD, and dyscalculia have garnered significant media and scientific attention over the past fifty years. This reflects a broader shift in medicine from merely ensuring survival to enhancing well-being and quality of life. Recognizing the multifactorial origins of many disorders, which often begin early in life, the DSM-5 introduced a new category called “Neurodevelopmental disorders”. These disorders are characterized by an early onset and a neurobiological basis, with adverse events during gestation or birth often playing a role. These include neurodevelopmental disorders (NDDs), which include intellectual disability, specific learning disabilities like dyslexia, dyscalculia, ADHD, and autism spectrum disorders (ASDs), as well as mental health disorders (MHDs), which include conduct, depression, anxiety, stress-related, and psychotic disorders. These illnesses affect 10–20% of children worldwide and are becoming more common [1]. NDDs have a major impact on children’s morbidity, impacting not only the individual but also the family and the community [2]. Children with dyscalculia, a form of learning disability related to numbers and mathematics, may struggle with numerical concepts, affecting their academic performance and overall well-being. Neurodevelopmental disorders, which begin in the developmental period and are lifelong, include conditions such as intellectual disability, ADHD, and ASD. They present a range of impairments affecting personal, social, academic, or occupational functioning, with diverse clinical and genetic profiles. Recent research emphasizes the importance of genetic links, neurobiological variations, and the need for targeted interventions, particularly for those within the criminal justice system. An improved understanding of these disorders’ biological mechanisms is crucial for developing effective treatments and support systems. Annie Swanepoel et al. [3] explore ADHD and ASD from an evolutionary perspective, suggesting that traits linked to these disorders may have conferred advantages in past environments but become maladaptive due to modern societal changes. They highlight the importance of considering both genetic and environmental factors, noting that ADHD may have favored risk-taking in hunter–gatherer societies, while high-functioning ASD could have been beneficial for developing specialist skills [4]. 

Suicidal ideation is significantly more likely in those with behavioral, psychotic, stress-related, depressive, and anxiety disorders [5,6,7,8]. Furthermore, there is a great deal of overlap between MHDs and NDDs; for example, ASD and ADHD frequently co-occur with anxiety and depression [9,10], while ADHD and depression frequently co-occur with depression [11,12]. These comorbidities can worsen learning disabilities in children and impact their quality of life [13], highlighting the importance of tailored treatments [14].

The development of these illnesses is influenced by both environmental and genetic factors. Poverty and poor family income are examples of socioeconomic circumstances that can have a significant impact on a child’s neurological functioning and mental health. These circumstances can also have an adverse effect on the child’s education, health, and self-esteem [15]. Furthermore, a parent’s incapacity to offer a nurturing atmosphere for their child’s development—a critical component in averting mental health issues—may be impeded by their own health difficulties, mental illnesses, social dislocation, or unstable housing.

Childhood-onset mental disorders, particularly NDDs, have profound effects on individuals, families, and society, underlining the urgency to understand and address these conditions. With prevalent NDDs like ADHD, dyslexia, and ASDs, this systematic review seeks to investigate how AI technologies have been used to support students. Additionally, it looks for ways to pinpoint the shortcomings of existing AI systems in order to guide future advancements and improve individualized learning for impacted children. In order to support children with NDDs and other mental health illnesses and help them reach their full potential, it is critical to identify and meet their requirements. The role of genetic and neurobiological factors in shaping reading and mathematical abilities is discussed in the research paper [16]. They highlighted how genetic variations influence the development of neural circuits, which are critical for the brain systems that facilitate learning in these domains. This pathway explains the interaction between genetic predispositions and environmental influences in the manifestation of dyslexia and dyscalculia. Andreas Demetriou et al. [4] propose a developmental theory integrating typical and atypical development, emphasizing central cognitive mechanisms and domain-specific processes. They highlight how deficiencies in these processes lead to various neurodevelopmental disorders, stressing the importance of early diagnosis and tailored interventions. Dyscalculia affects mathematical symbol manipulation and number recall due to deficits in coding numerosity, impacting arithmetic abilities and brain networks. Aphantasia involves the inability to create mental images, potentially linked to altered visual processing and the default mode network.

### 2.1. Historical Background of Dyscalculia

Dyscalculia, a specific learning disorder marked by difficulties in understanding numbers and mathematical concepts, has gained recognition only recently. Early descriptions of arithmetic difficulties date back to the late 1800s, but the term “dyscalculia” and a formal understanding of the disorder emerged much later. In the 20th century, researchers began to notice that some children, despite normal intelligence and educational opportunities, struggled specifically with math. Recent advancements in cognitive neuroscience and neuroimaging have significantly advanced the understanding and diagnostic criteria of dyscalculia.

### 2.2. Neurodevelopmental Disorders Today

The incidence of neurodevelopmental disorders, including dyscalculia, appears to be increasing, likely due to heightened awareness and improved diagnostic processes. Dyscalculia often coexists with other learning disorders like dyslexia and ADHD, complicating diagnosis and intervention. In the USA, increased attention to special education has led to more children being identified and supported, though the system still faces challenges in providing consistent and effective interventions. Three to seven percent of all children, adolescents, and adults suffer from dyscalculia. This figure corresponds to some 84,000 to 195,750 primary school pupils in Germany alone. The significance of dyscalculia is often underappreciated, yet poor mathematical ability places a major burden on both society and the affected individuals. A large-scale cohort study in England revealed that poor mathematical ability is associated with major psychosocial and economic risks: 70–90% of affected individuals ended their schooling prematurely at age 16, and at age 30, very few were employed full-time. Their probability of being unemployed and developing depressive symptoms was twice as high as those without dyscalculia. The annual cost arising from the severe impairment of mathematical ability in Great Britain is estimated at £2.4 billion.

### 2.3. Disorder of Attention Deficit Hyperactivity

Impulsivity, hyperactivity, and inattention are hallmarks of attention deficit hyperactivity illness (ADHD), a neurodevelopmental illness that affects people from childhood into adulthood. The frequency of the condition varies from 9% to 40%, depending on a mix of environmental and genetic factors, such as access to certain environmental chemicals, brain injury, preterm birth, and mother substance use during pregnancy. Comorbid conditions such oppositional defiance and conduct disorders, anxiety and depression disorders, and particular learning problems are common in children with ADHD. They struggle with sustained attention, hyperactivity, and participating in turn-taking activities. According to preliminary studies, children with and without ADHD have different brain sizes, especially in the frontal and parietal cortices, which may be a factor in the disorder’s behavioral and cognitive symptoms.

### 2.4. Dyslexia

Dyslexia stands out as a prevalent form of learning disability, impacting a significant portion, approximately 3% to 15%, of school-age children. Those affected by dyslexia encounter specific challenges in developing proficient reading skills, grappling with issues related to accurate and fluent word recognition, as well as difficulties in spelling and decoding. Studies highlight the functional brain imaging differences that can be seen between dyslexics and non-dyslexics, including a decreased neural response to repetitive stimuli. Furthermore, children diagnosed with dyslexia may contend with additional learning deficits alongside experiences of diminished self-confidence, heightened anxiety, and depression. Early detection and intervention remain pivotal in providing tailored support to individuals with dyslexia, empowering them to navigate hurdles and attain academic achievements. Establishing environments of support and fostering understanding among educators, family members, and peers are fundamental in nurturing the strengths and capabilities of those with dyslexia.

### 2.5. Spectrum Disorders in Autism

About 2% of people in affluent nations suffer from autism spectrum disorders (ASDs), which usually appear in the first three years of life. Autism spectrum disorders (ASDs) are characterized by challenges with social interactions, delayed speech and language, avoidance of eye contact, difficulty responding to changes in the environment, repetitive activities, and individual learning profiles. Anxiety and depression are common conditions among people with ASDs. The brain structure and function of people with ASDs differ from those of neurotypical people, according to research on the neurobiology of ASDs. For example, children with ASDs have been shown to have more neural connections in their brains than neurotypical people do. This finding suggests that during brain development, there may have been less pruning of faulty neuronal connections. These neurological variations lead to dysregulation in the coordination of cognitive functions across various brain regions as well as abnormal neural architecture across the brain. Comprehending these neuropathological pathways is essential to creating therapies and support plans that work for people with ASDs.

### 2.6. Dyscalculia

Mathematical comprehension and manipulation difficulties are the hallmark of a particular kind of learning disorder known as dyscalculia. It persists despite adequate education and intelligence, impacting academic performance and daily activities requiring numerical skills. Its exact causes involve genetic, neurological, and environmental factors, leading to structural and functional differences in the brain’s numerical processing areas. Early identification and tailored educational strategies, including multisensory approaches and explicit instruction, are crucial for supporting individuals with dyscalculia. Creating supportive environments and raising awareness about dyscalculia can help individuals affected by this learning disorder unlock their potential and succeed in mathematical learning and everyday tasks.

#### 2.6.1. Comorbidities

Children with dyscalculia frequently experience comorbid conditions, such as dyslexia or ADHD, complicating their educational and social experiences. Effective management requires a comprehensive approach that addresses all concurrent disorders. For instance, individuals with dyscalculia often have marked persistent problems in applying basic arithmetic methods and in the knowledge of math facts, which are not merely due to low intelligence or inadequate schooling. The prevalence of comorbid mental disorders is high among individuals with dyscalculia. They are at elevated risk of having dyslexia (odds ratio: 12.25), attention deficit hyperactivity disorder (ADHD), and other mental disorders, both internalizing (such as anxiety and depression) and externalizing (such as aggression and rule-breaking). Without specific intervention, dyscalculia often leads to scholastic failure and school absenteeism.

#### 2.6.2. Future Perspectives

Understanding dyscalculia and other neurodevelopmental disorders requires a long-term developmental perspective. Longitudinal studies are essential to track the progression of the disorder and the effectiveness of various interventions over time. There is a need for more research into the specific neurobiological mechanisms of dyscalculia and how they interact with other cognitive functions. Integrated and flexible educational strategies that go beyond rote learning and address underlying cognitive deficits are crucial. Schools and healthcare providers must work together to create personalized education plans that adapt to the evolving needs of children with dyscalculia, ensuring they receive the support necessary to succeed academically and socially.

## 3. Challenges of Prevalence Studies

The International Consensus describes developmental dyscalculia (DD) as a heterogeneous condition that causes differences in the development and performance of numerical cognition. This definition is supported by evidence from neuroanatomical, neuropsychological, and behavioral studies as well as their interconnections [9]. Based on its fundamental causes, DD is currently classified into primary or secondary categories. Primary developmental disorder (primary DD) affects 1–2% of school-age children and is characterized by particular, significant impairments in numeracy skills without other difficulties [17,18,19]. In contrast, roughly 4% of cases are secondary DD. When it comes to the person’s age or educational attainment, “non-numerical” cognitive deficiencies are just as severe as numerical impairments in secondary DD [20,21]. As an illustration, attentional deficiencies have been found to be a key cognitive marker of secondary DD in recent studies [17]. According to ICD-11 recommendations [20], secondary DD may also co-occur with other neurodevelopmental disorders as dyslexia or attention deficit hyperactivity disorder [22].

## 4. Understanding Dyscalculia: Identifying Its Characteristics, Causes, and Therapeutic Approaches

Dyscalculia has historically been defined as difficulties remembering math information and a continuous use of less sophisticated calculation techniques [23]. However, a growing corpus of recent behavioral and neuroimaging research suggests that dyscalculia may be caused by abnormalities in a neurobiological system that processes numerical magnitudes, or the total number of elements in a set. Recalling mathematical knowledge becomes challenging due to this impairment, which develops during learning and development. However, there are also disagreements on the role that larger cognitive processes like working memory and spatial attention play in the development of dyscalculia. The underlying cognitive processes related to dyscalculia are domain-specific, meaning they are particularly associated with numerical and mathematical cognition rather than a broad spectrum of cognitive functions.

### 4.1. Cognitive Processes Involved in Dyscalculia

#### 4.1.1. Number Sense

Approximate Number System (ANS): This involves an intuitive understanding of quantity and the ability to compare numerical values without counting. Individuals with dyscalculia often struggle with basic estimations and numerical comparisons, indicating a deficit in ANS.

Symbolic Number Representation: Understanding that symbols (e.g., digits) represent quantities is a fundamental aspect. Dyscalculia can impair the ability to link symbols with their respective quantities.

#### 4.1.2. Working Memory

Working memory is crucial for holding and manipulating numerical information. Individuals with dyscalculia often have difficulties retaining intermediate steps in calculations, following multi-step problem-solving procedures, and recalling arithmetic facts.

#### 4.1.3. Visuospatial Skills

These skills involve the ability to visualize and manipulate objects and are essential for understanding spatial relationships in geometry, reading graphs, and comprehending the arrangement of numbers. Dyscalculia can lead to problems with spatial organization of numbers, leading to frequent errors in calculation layout and number alignment.

#### 4.1.4. Executive Functions

Executive functions, including planning, problem-solving, and task switching, play a critical role in mathematical reasoning and the application of strategies. Dyscalculia often involves deficits in these areas, making it difficult to plan multi-step problems or switch between different mathematical strategies effectively.

#### 4.1.5. Attention

Sustained attention and the ability to focus on numerical tasks are necessary for performing calculations and solving problems. Individuals with dyscalculia may have difficulty maintaining attention on math-related tasks, leading to mistakes and incomplete work.

#### 4.1.6. Language Processing

Understanding and processing mathematical language, such as word problems, instructions, and mathematical terminology, are essential. Dyscalculia can affect the ability to comprehend and process this language, further hindering mathematical performance.

### 4.2. Domain-Specific Factors

Numerical Processing: Dyscalculia is primarily characterized by impairments in the processing of numerical information, such as difficulties with basic arithmetic operations, number comparison, and understanding mathematical concepts.

Mathematical Fact Retrieval: Difficulty in retrieving basic math facts (like multiplication tables) from long-term memory is common in dyscalculia. This can lead to slow and laborious calculations.

Procedural Memory: Problems with learning and remembering mathematical procedures, like long division or multi-step equations, are often observed.

### 4.3. Arithmetic

Dyscalculia (DD) is primarily characterized by impaired arithmetic fact retrieval, typically observed in early grades where typically developing children transition from procedural to memory-based calculation strategies. While their peers advance, children with DD frequently struggle to acquire fluent fact-retrieval processes and instead stick to procedural methods. They recall significantly fewer arithmetic facts compared to typically developing children, indicating the severity of their deficit. This difficulty in fact retrieval leads to the use of immature problem-solving strategies, such as counting all, which are inefficient. The manifestation of math difficulties varies over time, with some children showing deficits from kindergarten onwards, impacting later skill acquisition. Persistent deficits are crucial for diagnosing DD accurately, as these challenges may evolve over time. While impaired fact retrieval and strategy use define DD, they are also present in individuals with secondary DD/MLD, complicating diagnosis. According to some researchers, primary DD could be caused by a fundamental impairment in number sense, which has an impact on how numerical magnitudes are represented and processed. The role of number sense in children with Down syndrome is being investigated in an ongoing study. Researchers asked participants to complete two rounds of a task involving checking single-digit addition problems in order to learn more about how people handle arithmetic issues [24]. In this activity, half of the trials presented with proper solutions, and participants had to decide if the solution presented could solve the problem or not. All information was presented in white against a black background. Three circumstances were included in the task: “plus1” issues large problems (where the solution was greater than 10), and small problems (where the solution was 10 or less). None of the problems that produced ties or used zero as an operand were included. The task consisted of 36 rounds with 12 problems for each condition [25].

### 4.4. Basic Number Processing Arithmetic

Research suggests that children with mathematical learning difficulties (MLD) may not exhibit the same automatic activation of numerical magnitude information as typically developing peers. This reduced automaticity in processing numerical information has been supported by various studies, including those employing the numerical comparison paradigm. Such studies indicate impaired performance in numerical comparison tasks among individuals with MLD [26]. Additionally, these individuals tend to display a larger numerical distance effect (NDE), suggesting less precise or noisier representations of numerical magnitude. It has also been noted that different MLD patients have varying degrees of defective numerical magnitude processing; main DD may be associated with more severe arithmetic deficiencies as well as a genetic impairment in representing and processing numerical magnitudes. On the other hand, secondary DD may be linked to less severe math problems that are unrelated to problems processing numerical magnitudes. This emphasizes how crucial it is to differentiate between primary and secondary DD in order to comprehend the etiology and severity of their phenotype.

### 4.5. Non-Numerical Shortcoming

Some researchers suggest that dyscalculia (DD) may arise from issues with broader cognitive functions like working memory, visual-spatial processing, or attention. While some studies support this, others with stricter criteria found no differences in working memory between children with and without mathematical difficulties. Research demonstrating that children with DD score worse on attention and visual-spatial processing tests has led to the notion that deficiencies in visuospatial attention are the root cause of DD. Additionally, some suggest a disruption in linking Arabic digits with numerical magnitudes as a root cause of DD, backed by evidence of impaired symbolic numerical processing. Neuroimaging research indicates the involvement of specific brain regions in numerical magnitude representation, possibly underpinning DD. To completely understand the relationship between these cognitive processes and DD, more research is necessary.

### 4.6. Neural Characteristics of Dyscalculia

Neural characteristics pertain to the underlying brain features associated with dyscalculia (DD), a specific learning disorder affecting mathematical abilities. This area of study focuses on identifying differences in brain structure and function between individuals with DD and those without. Certain brain regions implicated in DD can be identified with the use of neuroimaging techniques including structural magnetic resonance imaging (MRI) and functional magnetic resonance imaging (fMRI). For instance, the intraparietal sulcus (IPS), crucial for processing numerical magnitude, shows variations in structure or activity among those with DD. These neural traits offer insights into the mechanisms underlying DD and guide the development of interventions to address this learning challenge [27,28,29].

### 4.7. Matching

Basic number processing was tested using a matching task that evaluated the basic semantics that include symbolic and non-symbolic numerical values [25,30]. Participants in a particular condition saw a number sign along with a series of dots. They had to decide if the two representations showed the same amount (which happened in 50% of trials). In contrast, participants had to determine whether two shapes—such as a circle and a star—were the same (which happened in 50% of trials) or dissimilar in the shape condition. In addition, facial matching was included as a third criterion. Preliminary testing revealed differences in difficulty between the number and shape conditions, therefore this condition was adjusted to meet the number condition’s difficulty level. Participants were presented FaceGen Artist-created pairs of Asian faces facing forward in this situation. Participants were asked to determine if the faces matched the same individual. Each participant completed two runs, with two blocks and a total of six trials (sixteen trials total for each condition) for each condition. Before every block, a cue displaying an example stimulus was displayed.

### 4.8. Visuospatial Working Memory

A task adapted by Dumontheil and Klingberg was used to study working memory. Participants saw a red dot moving over a white 4 × 4 grid on a black background in the experimental arrangement. The red dot disappeared, and in place of it was a blank red circle. In 50% of trials, participants were instructed to press a button to indicate whether or not the location of the empty red circle matched the prior location of the red dot. In contrast, under the control condition, participants saw a blue dot move across the grid. Participants were instructed to use their right hand to click the button as soon as the goal stimulus—an empty blue circle—appears, regardless of where it is positioned. Both scenarios included trials with low load (the dot passing through three grid locations) and high load (the dot passing through five grid locations). Every participant finished two runs, each of which included six trials for each of the two load levels in every condition. These activities were all given in block designs, with a 6500 ms start fixation block and a 12,000 ms final fixation block coming first. Six trials of a particular condition were included in each block, with an average jittered inter-trial interval (ITI) of 1500 ms. In the matching task, stimuli were exhibited for 2000 ms, whereas each problem in the arithmetic task was displayed for 4500 ms. The trial length for the Visual-Spatial Working Memory (VSWM) test changed according to load. For 500 ms, each dot location was displayed; this was followed by 500 ms of a blank grid. Following the dot traversal, the target screen appeared for 1500 ms, followed by a waiting screen. Throughout the ITI, participants were told to answer precisely and on time, even after the stimulus vanished. Their responses were also timed. Latin square counterbalancing was used to ensure that each participant completed the trials in a randomized order in order to minimize order effects. Blocks (i.e., 6, 9, or 12 s) were separated by an average of 9 s. E-Prime 2.0 (Psychology Software Tools, Pittsburgh, PA, USA) was used to present all of the exercises, and participants responded by clicking a button on a response box. Stimuli were projected onto a screen at the end of the scanner bore using a mirror attached to the head coil. Those whose accuracy in any combination of conditions and runs was less than 50% were excluded from the analysis for that test. Moreover, those exhibiting 0% accuracy on a given condition were excluded from all analyses pertaining to that specific task. These exclusion criteria led to the elimination of three children (one TA and two DD in the arithmetic task), four in the VSWM task (one TA and three DD), and none in the matching task.

## 5. Treatment for Dyscalculia across Different Ages and Disorder Profiles

The effective treatment for dyscalculia varies by age and specific disorder profiles. For young children (ages 3–6), play-based learning using numerical games and manipulatives, interactive storybooks, and parental involvement in daily counting activities can help develop basic numerical understanding. Elementary school children (ages 7–12) benefit from structured math instruction, using the Concrete–Representational–Abstract (CRA) approach, educational software [28], memory aids, and tutoring.

Adolescents (ages 13–18) with advanced mathematical reasoning deficits can use executive function training, assistive technology like graphing calculators, peer tutoring, and accommodations such as extended test times. Adults often need functional math instruction related to daily tasks and workplace skills, cognitive–behavioral therapy for math anxiety, continued education, and assistive technology for financial management and planning.

For individuals of any age with co-occurring disorders like ADHD or dyslexia, integrated approaches involving multidisciplinary support, behavioral interventions, language support, and training for parents and teachers are essential. Tailoring interventions to the individual’s age and specific needs ensures more effective support for overcoming dyscalculia challenges.

## 6. Role of Technologies in Special Needs Education

The job landscape for persons with disabilities is evolving quickly as a result of technological advancements like machine learning (ML), artificial intelligence (AI), the Internet of Things (IoT), and other developments. A broad spectrum of children and young adults with numerous disabilities, such as difficulties with reading and writing, difficulties with social interaction, comprehension problems, and more, are included in the term Special Education Needs and Disabilities (SEND). According to data from the National Statistical Institute of Bulgaria in 2017, 4.7 out of 1000 Bulgarian children under the age of sixteen have a disability.

Technology has changed many facets of daily life in the last ten years, and this has had a significant influence on our understanding of human intellect. Computer science efforts have focused on artificial intelligence, trying to develop intelligent agents that can sense their surroundings and make choices that will increase their chances of success. Education intersects with AI as a subfield, with numerous scientists and experts acknowledging the positive potential of AI technologies in education to cater to diverse learning needs.

The emphasis on offering inclusive education to all children, adolescents, and adults has grown in recent years, highlighting the expanding use of technology to solve learning issues. AI’s educational benefits have long been recognized, extending into special education where expert systems were initially employed to mimic human expertise in intellectual tasks.

The evolution of artificial intelligence has significantly impacted higher education services, exemplified by IBM’s supercomputer Watson, already utilized by colleges as an early AI iteration. Deakin University in Australia, for instance, offers round-the-clock assistance from Watson, highlighting its transformative potential in administrative functions, such as timetable management and service quality, potentially altering staffing dynamics.

Machine learning, a promising AI branch, differs from traditional systems by its ability to learn patterns and anticipate outcomes. For example, the AlphaGo software (https://deepmind.google/discover/blog/alphago-zero-starting-from-scratch/ accessed on 14 May 2024) developed by Google’s DeepMind AI division beat the best Go player in the world, proving machine learning’s ability to identify patterns, predict the future, and adjust to novel situations that AI has never encountered before.

### AI for Learning Disabilities

Although the definition of artificial intelligence (AI) has been proposed for decades, the field is still at odds. As a result, definitions of artificial intelligence are numerous and differ according to the sector. Samoili et al. [31] carried out a qualitative analysis of more than 50 texts defining AI in order to shed light on the concept. A high-level expert committee then used the analytical results to produce an operational definition for AI. Artificial Intelligence (AI) is defined as human-designed hardware and software that function in the digital or physical world by observing its surroundings through information processing, interpretation, reasoning, and data collection, and then deciding on the best course of action to accomplish a given objective.

Over the past few decades, the popularity of AI has fluctuated, and machine learning is a subfield that has recently attracted a lot of attention from both academia and industry (Thompson et al. [32].). This is a significant development because it permits the computer to “learn” from “experience” and use what it has discovered to tackle new challenges, much like people do. Due to the overwhelming interest in this new feature, various AI applications and technologies (such as computer vision and natural language processing) are being created and used in every sphere of society, including education (Zhai et al. [33,34,35,36]). These forms include intelligent tutors, chatbots, communication aids, adaptive learning devices, intelligent robots, mastery learning devices, and facial recognition software. Due to the diversity in the nature and scope of AI applications in education, AI is a potent instrument for identifying and providing appropriate help for the particular difficulties faced by SWLDs. In recent years, there have been an increasing number of publications in the literature discussing the application of AI to improve learning outcomes for students with learning challenges. In a review study [23], author discussed the usage of machine learning apps for dyslexia prediction as well as e-learning for learning and cognitive impairments. To improve learning, external AI-based appearances were utilized in six out of the twenty-four tests that were examined. Specifically, four of them focused on providing personalized or tailored education, one on the effects of virtual learning, and one on machine learning treatments in general. The bulk of the studies that were analyzed (*n* = 13) concentrated on diagnosing, predicting, or screening for learning disabilities or difficulties. As to the research conducted [23], a notable focus of AI research for SWLDs has been on diagnosing, screening for, or predicting learning problems. The most crucial, but intricate, area of study has received less attention: enhancing learning for SWLDs.

In contrast to study [23], this literature review focused on research that used AI to support SWLDs in ways other than learning disability diagnosis, screening, or prediction.

## 7. Tests to Identify Dyscalculia

Several tests are available specifically designed to assess dyscalculia. Some commonly used tests include the following:

Dyscalculia Screener: This screening tool is specifically designed to identify indicators of dyscalculia in children and adults. It assesses various aspects of numerical processing and mathematical abilities.

Developmental Dyscalculia Diagnostic Test (DDDT): The DDDT is a comprehensive assessment tool that evaluates mathematical abilities and identifies specific deficits related to dyscalculia.

The Basic Number Skills subtest of the Dyslexia, Dyspraxia, and Dyscalculia Test (DDDT-BNS): This subtest assesses basic numerical skills, including counting, number recognition, and simple calculations, which are often impaired in individuals with dyscalculia.

TEDI-MATH (Test for the Diagnosis of Early Number Skills–Mathematical Disabilities): This test is designed to assess early numerical skills and identify difficulties related to dyscalculia in young children.

The Dyscalculia Check: This assessment tool evaluates mathematical abilities across different domains, including numerical reasoning, arithmetic fluency, and spatial skills, to identify potential dyscalculia.

The Dyscalculia Toolkit: This comprehensive assessment resource includes various tests and checklists to evaluate mathematical abilities and identify dyscalculia in children and adults.

Kay Math Diagnosis Test: Kaufmann and von Aster [37] provide a comprehensive overview of dyscalculia diagnosis and management, discussing assessment methods like the Key Math Diagnostic Test and outlining intervention strategies tailored to individuals with dyscalculia.

It is essential to note that these tests should be administered and interpreted by qualified professionals, such as educational psychologists or specialists in learning disabilities, to ensure accurate diagnosis and appropriate intervention. Additionally, assessments for dyscalculia often involve a combination of standardized tests, clinical interviews, observations, and other evaluation methods to provide a comprehensive understanding of an individual’s mathematical difficulties.

The workplace landscape is swiftly evolving for individuals with disabilities, driven by technological advancements like the IoT, AI, ML, and other innovations.

### 7.1. Diagnostic Tools for Dyscalculia

Accurate diagnosis of dyscalculia requires tools that evaluate both domain-specific and domain-general cognitive processes. Here are some effective diagnostic tools.

#### 7.1.1. Domain-Specific Diagnostic Tools

Numerical Processing Tests: Number Sense Assessments evaluate the understanding of numerical magnitude, quantity comparison, and basic arithmetic operations.

Symbolic Number Representation Tests: These tests assess the ability to connect symbols (e.g., digits) to their respective quantities and perform operations.

Arithmetic Achievement Tests: Standardized tests like the Wide Range Achievement Test (WRAT) and the Test of Early Mathematics Ability (TEMA) measure specific math skills.

Mathematical Fact Retrieval Tests: These tests assess the speed and accuracy of recalling basic math facts, such as multiplication tables and addition/subtraction facts.

Procedural Memory Assessments: Tasks that require solving multi-step mathematical problems to evaluate the ability to remember and execute procedural steps.

#### 7.1.2. Domain-General Diagnostic Tools

Working Memory Tests: Digit Span Tests measure the ability to hold and manipulate numbers in working memory. The Corsi Block-Tapping Test (CBT) evaluates visuospatial working memory.

Visuospatial Skills Assessments: The Rey–Osterrieth Complex Figure Test assesses the ability to reproduce complex geometric shapes. The Block Design Test (from the WISC or WAIS) measures spatial visualization and problem-solving skills.

Executive Function Tests: The Wisconsin Card Sorting Test (WCST) assesses cognitive flexibility and problem-solving abilities. The Tower of London Test measures planning and problem-solving skills.

Attention Assessments: The Continuous Performance Test (CPT) measures sustained attention and response control. The Trail Making Test evaluates attention, sequencing, and task-switching capabilities.

Language Processing Tests: The Clinical Evaluation of Language Fundamentals (CELF) assesses the understanding and processing of language, including mathematical terminology.

### 7.2. Treatment Tools for Dyscalculia

Effective treatment for dyscalculia should address both domain-specific and domain-general cognitive processes through tailored interventions and support.

#### 7.2.1. Domain-Specific Treatment Tools

Structured Math Instruction: Explicit teaching methods involve direct and systematic instruction focusing on specific math concepts and procedures.

Concrete–Representational–Abstract (CRA) Approach: Helps students understand mathematical concepts through hands-on activities, visual representations, and abstract symbols.

#### 7.2.2. Technology-Based Interventions

Educational Software and Apps: Tools like Khan Academy, Mathseeds, and NumberShire provide interactive and adaptive math practice.

Math Games: Engaging games that reinforce mathematical concepts and improve numerical fluency.

#### 7.2.3. Multisensory Techniques

Manipulatives: Use physical objects like counters, blocks, and number lines to visualize and solve math problems.

Visual Aids: Diagrams, charts, and visual organizers to help understand and retain mathematical information.

#### 7.2.4. Memory Aids

Mnemonics and Rhymes: Techniques to help memorize math facts and procedures.

Flashcards: Repeated practice with basic math facts to improve retrieval speed.

### 7.3. Domain-General Treatment Tools

#### 7.3.1. Working Memory Training

Cognitive Training Programs: Tools like Cogmed and Lumosity offer exercises to improve working memory capacity and function.

Chunking Techniques: Breaking information into smaller, manageable chunks to aid memory retention.

#### 7.3.2. Visuospatial Skills Development

Spatial Reasoning Games: Puzzles and activities that enhance spatial awareness and visualization skills.

Drawing and Construction Activities: Tasks requiring constructing shapes or visualizing objects in space.

#### 7.3.3. Executive Function Interventions

Problem-Solving Strategies: Teaching step-by-step approaches to tackle complex problems.

Self-Monitoring Techniques: Encouraging students to check their work and reflect on their problem-solving process.

#### 7.3.4. Attention Training

Mindfulness Practices: Techniques to improve focus and attention span, such as mindfulness meditation and deep-breathing exercises.

Task Structuring: Breaking tasks into smaller steps and providing clear, structured instructions.

#### 7.3.5. Language Support

Vocabulary Building: Teaching and reinforcing mathematical terminology and language.

Reading Comprehension Strategies: Techniques to improve understanding of word problems and mathematical instructions.

### 7.4. Integrated Approaches

#### 7.4.1. Individualized Education Plans (IEPs)

Tailored IEPs that address both domain-specific and domain-general needs, involving educators, parents, and specialists.

#### 7.4.2. Collaborative Interventions

Multidisciplinary teams, including educators, psychologists, occupational therapists, and speech-language pathologists, providing comprehensive support.

#### 7.4.3. Parental and Teacher Training

Educating parents and teachers on effective strategies to support children with dyscalculia, including how to use assistive tools and techniques at home and in the classroom.

By combining these diagnostic and treatment tools, educators and clinicians can provide targeted and effective support to individuals with dyscalculia, addressing both their specific mathematical difficulties and broader cognitive challenges.

## 8. Drawbacks of Existing System

A thorough evaluation tool, the Woodcock–Johnson Tests of Achievement (WJ ACH) is intended to test academic achievement throughout an extensive age range (2–95 years old) and grade levels (K.0–18.0). It consists of 22 subtests that include reading, math, writing and vocal language, knowledge, and five major areas of academic accomplishment. While the extended battery has 14 subtests with the option to add more to get extra points, the basic battery only has seven subtests. The math battery, which is especially helpful for monitoring development in the reading, writing, and math accomplishment domains, is the subject of this study.

The math battery assesses various skills essential for mathematical calculations ranging from basic arithmetic to trigonometry. Mathematical problems including multiplication, division, fractions, decimals, basic algebra, and other topics are given to test takers. Some subtests, such as Math and Calculations [Test 5], Applied Problems [Test 10], Quantitative Concepts [Test 18A], and Number Series [Test 18B], are not timed; however, the Math Fluency [Test 6] test is. Examiners assess scores according to performance, grade, and age in addition to examining the kinds of errors made. For instance, errors like 94 − 37 = 67 may indicate difficulties in grasping fundamental mathematical concepts like carrying over and borrowing. However, some mistakes may suggest attention-related issues, such as misidentifying the operation involved in a problem.

The Wechsler Intelligence Scale for Children (WISC III and IV) is administered to assess children’s intellect, consisting of verbal and performance sections. Scores above 130 are considered excellent, while scores between 120 and 129 are high, between 110 and 119 are moderate, and below 90 are average. A score of less than 70 denotes borderline mental functioning, whereas a score of less than 69 denotes mental retardation.

An additional screening tool for determining whether a more thorough accomplishment evaluation is necessary is the Wide Range Accomplishment Test (WRAT). It assesses reading, math, and spelling abilities using timed exams that include both verbal and numerical questions.

For diagnosing and assessing dyscalculia, psychoeducational assessments and curriculum-based tests are commonly used. However, the process can be challenging due to the varying profiles of arithmetic skills and the lack of standardized processing tests for the Indian children’s population. Curriculum-based tests may not fully capture all deficits and can lead to an overdiagnosis of dyscalculia. Hence, a more accurate diagnostic tool tailored to Indian children’s needs was deemed necessary to improve the assessment and diagnosis of dyscalculia. A number of tests, including the Kaufman Test of Educational Achievement (KTEA), Woodcock–Johnson Tests, Wechsler Individual Achievement Test (WIAT), Wide Range Achievement Test (WRAT), and the Grade Level Assessment Device (GLAD), have been created to identify specific learning disorders (SLDs). These tests are effective in identifying SLDs by analyzing the scores attained. To ensure accurate findings, extra curriculum-based tests (CBTs) may be necessary in some situations to supplement these tests. The manual diagnosis process using these tests can be tedious, leading to many cases of SLDs going undiagnosed, despite affecting approximately 3–7% of the global population, particularly dyscalculia.

Given the complexity and variability of assessment tests, traditional diagnosis methods have been cumbersome. Therefore, this study aims to automate the screening process using machine learning algorithms, which have shown promise in predicting outcomes based on input parameters. Previous applications of machine learning in education have focused on predicting student performance and determining optimal teaching methods. Machine learning techniques offer the advantage of automatic learning from complex data patterns, enabling the development of models capable of identifying dyscalculia.

Early symptoms of dyscalculia, such as difficulties in basic counting, understanding quantities, and arithmetic skills, can manifest in children as young as 6–7 years old. Timely intervention is crucial for addressing these deficits. Research has shown that computer-based interventions can enhance mathematical understanding and motivation among children with dyscalculia. However, existing interventions often have limitations, such as language barriers, platform dependence, and age restrictions.

Although learning disorders have been diagnosed using machine learning algorithms, there are not many research studies that concentrate on dyscalculia screening specifically. Furthermore, existing interventions often fail to address the needs of children at the critical age of 6–7 years when symptoms first appear. Thus, it is necessary to create machine learning models that can test kids this age and get around the drawbacks of the screening instruments that are now in use.

## 9. Literature Survey

A rising number of people are interested in employing artificial intelligence (AI) technology to improve special needs education (SNE), according to Neeharika et al. [38]. This interest is centered on the potential advantages of AI for SNE students, as well as its function in supporting teachers. Studies also emphasized the use of data-driven methods, namely in the categorization of participants who are normally developing (TD) and individuals with autism spectrum disorder (ASD).

PD Barua et al. [39] highlight the rising prevalence and co-morbidity of neurodevelopmental disorders (NDDs) and mental health disorders (MHDs) in childhood. AI-assisted tools, utilizing machine learning, show promise in addressing learning challenges and improving social interaction for children with NDDs, though improvements in personalization are needed.

The research paper [40] discussed that dyslexia, dysgraphia, and dyscalculia are prevalent learning challenges affecting roughly 10% of children worldwide. Despite the growing tech-savviness in society, there is a noticeable gap in utilizing mobile apps for screening and addressing these disabilities in Sri Lanka. This study introduced “Pubudu”, an innovative mobile application using deep learning and machine learning techniques to identify and support these learning difficulties in local languages. The software uses machine learning for dyscalculia and deep neural networks for assessments of dyslexia and dysgraphia, in accordance with clinical guidelines. Engaging game-like activities are integrated for interventions. Promising test results were obtained from 50 children with learning disabilities and 50 without them: 88% accuracy for letter dysgraphia, 58% for dyslexia, 99% accuracy for numeric dysgraphia, and 90% accuracy for dyscalculia.

“Pubudu” holds significant potential in aiding children with these challenges, particularly benefiting disadvantaged children in Sri Lanka. Predicting math difficulties (MD) in Grade 6 early on is essential for providing timely help.

Psyridou et al. [41] used neural networks and information from kindergartens to create a model with 49 factors. These factors ranged from arithmetic and cognitive skills to the home environment, parental inputs, and behavior. The model performed well with an AUC of 0.818, highlighting its potential to identify MD early and offer timely support in schools. The neurological differences between children with developmental dyscalculia (DD) and their normally achieving classmates during number processing are examined by Karin Kucian et al. [42]. Dyscalculic children showed weaker activation in key brain regions like the left IPS, left IFG, and right MFG, crucial for accurate number processing. However, both groups generally utilized similar neural networks for number tasks, suggesting specific challenges in neural resource recruitment for analog number magnitudes in dyscalculic children. Future research is needed to understand effective training methods and bridge neuroscience findings with classroom practices for children with DD.

Kriti et al. [28] proposed a study which examines the effectiveness of the assistive technology tool, Mathlete, in enhancing early numeracy skills of children with dyscalculia. Out of 40 participants, 30 used Mathlete while 10 received traditional teaching. Children using Mathlete showed significant improvements in understanding mathematical concepts compared to those in the control group. Mathlete proved beneficial in enhancing mathematical learning for children with dyscalculia. Adriano et al. [43] discussed the study that uses artificial intelligence tools to comprehend how study techniques, learning difficulties, and academic achievement interact. The research identifies critical factors impacting academic achievement and suggests tailored educational interventions using artificial neural networks and a fuzzy AI. The results highlight how important it is to use specialized teaching strategies to lessen the effects of learning difficulties, foster inclusivity, and increase students’ academic achievement. Kaser et al. [44] presented an adaptive computer-based training system designed to improve mathematics learning in children with developmental dyscalculia or math difficulties. The system uses a dynamic Bayesian network student model to personalize learning by identifying appropriate tasks based on the student’s knowledge level. It evaluates student actions to extract patterns for predictive control, enhancing both learning success and motivation. The study includes comprehensive testing and pilot results demonstrating the effectiveness of the optimized training approach. Gupta et al. [45,46] explored dyslexia, a hereditary neurological condition affecting approximately 10% of individuals and leading to reading and writing challenges in about 20%. They emphasized the importance of early guidance in mitigating these challenges. This study introduced an Android application as a digital solution to assist dyslexic students, enhancing learning through practice in alphabets, numbers, and words, aligning with students’ preference for digital tools. Yaquob et al. [47] introduced DAELMS, an adaptive e-learning system leveraging artificial intelligence to cater to students’ learning styles and dyslexia types. It addresses the flexible nature of e-learning systems, emphasizing personalized online learning interventions beyond traditional computerized methods [48]. Flogie et al. [49] developed intelligent serious games to enhance social and cognitive competences in children with learning disabilities, demonstrating significant improvements in evaluations across participant groups. The research employed a 4-step methodology, incorporating needs analysis, content development, game creation, and usability evaluation to tailor learning experiences [38,39,50,51,52,53,54,55]. The machine learning techniques, including Bag of Features (BOF) and classifiers like SVM and Naïve Bayes, to predict student engagement in dyslexic learners using facial image analysis. The prototype, a Dyslexia Adaptive Learning Model, achieved high accuracy rates of 97–97.8% in distinguishing between engaged and disengaged states. Despite challenges in gesture differentiation, the model demonstrates potential for enhancing educational support for dyslexic students. Panjwani et al. [56] highlight seven AI applications designed to support students with learning disabilities: Adaptive Learning, Facial Expression, Chat Robot, Communication Assistant, Mastery Learning, Intelligent Tutor, and Interactive Robot [3,4,16,42,57] (Table 1).

## 10. Use of AI in Dyscalculia

### 10.1. Conventional Methods Using AI

**Figure 1:**
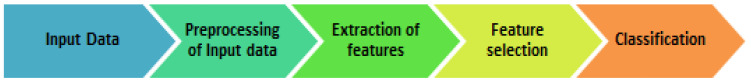
Sequence of steps using the machine learning algorithm.

One particular type of learning disability called dyscalculia is typified by problems with math abilities. Within the field of artificial intelligence (AI), machine learning functions by means of autonomous task execution, devoid of human involvement. Conventional machine learning models may generate precise predictions since they are trained on input data. Large datasets are utilized by deep learning, another subfield of machine learning, to attain high prediction accuracy. With remarkable accuracy, these models have been used to diagnose a number of neurological conditions, including dyscalculia. The use of computerized tomography (CT), MR imaging (MRI), PET (positron emission tomography) scans, and electroencephalogram, or EEG, signals can all provide diagnostic information for these types of illnesses. A model based on machine learning is trained by first receiving signals or images, then pre-processing the data to improve the quality of the information. Following this, characteristics have been extracted and sorted, which identify important aspects for categorization as normal or abnormal. Deep learning algorithms automate feature extraction and selection, in contrast to standard models that require human intervention. Figure 1 shows the sequence of steps involved in the machine learning algorithm.

### 10.2. Advanced Methods of AI

In the realm of dyscalculia research, advanced AI methodologies are gaining prominence. The important deep learning models used are the autoencoder, long short-term memory (LSTM), convolutional neural network (CNN), etc. In CNNs, input data undergo convolutional layer processing, creating successive feature maps that extract increasingly robust features for predictive purposes. The final layer classifies this data. LSTMs consist of three primary memory cell blocks: the input, forget, and output gates. These gates manage the storage, reading, and writing of information as new data arrive, focusing on retaining essential information from previous states. Autoencoders, on the other hand, are constructed from encoders that form a deeper model. They function by encoding unlabeled input data and accurately reconstructing them thereafter. These methodologies are instrumental in decoding and understanding the complexities of dyscalculia.

## 11. Dyscalculia Screening

Dyscalculia screening refers to the process of identifying individuals who may have dyscalculia, a specific learning disability that affects a person’s ability to understand, learn, and perform math and number-based tasks. The goal of screening is to detect signs of dyscalculia early so that appropriate interventions can be implemented to support the individual’s learning and development. Dyscalculia screening is a vital process in identifying and supporting individuals with mathematical learning difficulties. Advances in AI and adaptive technologies are enhancing the precision and effectiveness of these screenings, enabling earlier and more accurate identification, which is essential for providing the necessary educational support and interventions. Panjwani-Charania and Zhai et al.’s, [35] systematic review examines the use of artificial intelligence (AI) to support students with learning disabilities (SWLDs), focusing on applications beyond just diagnosis and identification. The review covers 16 studies, primarily on dyslexia and general learning disabilities, with half focusing on school-age children. It identifies seven AI applications: adaptive learning, facial expression recognition, chat robots, communication assistants, mastery learning, intelligent tutors, and interactive robots, with adaptive learning being the most common. Using the SAMR-LD model, the review categorizes AI integration into four levels: substitution, augmentation, modification, and redefinition. It finds that AI has been utilized at all levels to aid SWLDs, highlighting AI’s potential to enhance learning experiences by providing personalized support and adapting materials to students’ needs. Despite its promise, the review notes a lack of empirical studies and calls for more research. Effective AI integration is essential, as technology alone cannot improve learning outcomes. The SAMR-LD model illustrates how higher levels of AI integration can transform learning for SWLDs. The review concludes by advocating for further research on AI applications to support SWLDs, suggesting that AI could significantly improve academic performance and social-emotional well-being for these students.

Francesco Blangiardi et al. [58] present an AI system named PDEP_Evaluator. This AI is pivotal in generating and adapting trials for a non-symbolic number comparison game called The Number Farm, which educates players by presenting them with balanced difficulty levels according to their skills, based on the Learning to Focus on Number theory. The AI functions within the ND-NND space, where ND (numerical difference) determines the correct answer and NND (non-numerical difference) influences player perception. Players are modeled as linear classifiers, with decision-making influenced by parameters α (the angle representing the influence of NND) and σ (the standard deviation of noise, reflecting the sharpening hypothesis). The AI adapts to players by estimating these parameters from their responses, ensuring personalized and effective learning experiences. It outlines the development of an AI system for educational gaming, emphasizing its adaptive capabilities and theoretical foundation in enhancing numerical understanding through tailored trials.

Filipa Tinoco Ferraz et al. [59] discuss an innovative framework designed to address developmental dyscalculia (DD), a prevalent but under-researched learning disorder affecting mathematical abilities in 6% to 7% of the population. The framework focuses on screening students, adapting to their needs, and evolving with them, emphasizing the importance of a cognitive approach using current technologies for early intervention.

The research is a multidisciplinary effort involving university departments and external experts, aiming to formally understand dyscalculia in children, identify their difficulties, and analyze therapy outcomes. A deductive–inductive methodology is used, incorporating conceptual theory, practical solutions, and a game-based approach for therapy and diagnosis. The key component is disMAT, a game-based solution designed to be both engaging and educational, targeting specific difficulties faced by children with dyscalculia. This is supported by Discalculia Web, a web application for educators and experts to track progress and provide screening and therapeutic guidance. Technical details include an API, cognitive engine, database, and web server, all supporting the mobile and web applications. Initial tests in schools with children aged 6 to 11 have shown promising results in identifying and providing appropriate guidance for dyscalculia. It provides a comprehensive approach to understanding and addressing dyscalculia in children through a multidisciplinary research initiative and the development of an advanced, game-based learning support system.

### 11.1. Key Components of Dyscalculia Screening

The assessment of numerical abilities involves several key components for screening individuals’ mathematical proficiency. Basic Number Sense evaluates comprehension of numbers, counting, and their relationships, while Arithmetic Skills test the capacity to perform fundamental operations such as addition, subtraction, multiplication, and division. Number Line Estimation assesses one’s ability to estimate numerical values on a number line, particularly pertinent in dyscalculia assessments [60]. Cognitive functioning tests, including Working Memory and Attention/Executive Function, measure the capacities crucial for mathematical reasoning, such as information retention and problem-solving skills. Behavioral and developmental history collection involves gathering information on academic performance, developmental milestones, and reported math-related difficulties from teachers or parents. Standardized testing utilizes tools tailored for dyscalculia screening, offering benchmarks for comparison against expected age-related norms. These comprehensive evaluations aim to provide a holistic understanding of an individual’s mathematical abilities and potential challenges.

### 11.2. Advances in AI-Enhanced Screening

Adaptive Testing: AI can administer adaptive tests that change in difficulty based on the individual’s responses, offering a more personalized and accurate assessment.

Pattern Recognition and Data Analysis: Machine learning algorithms can analyze large datasets to identify patterns in errors and response times that are indicative of dyscalculia [58].

Neuroimaging and Biometrics: Advanced screening might include neuroimaging techniques to detect neurological markers associated with dyscalculia, providing a more objective assessment.

Interactive Digital Platforms: AI-driven platforms can offer interactive and engaging ways for children to complete assessments, reducing test anxiety and providing more accurate results.

### 11.3. Importance of Early Screening

Early detection of dyscalculia is crucial for several reasons:

Timely Intervention: Early identification allows for the implementation of targeted interventions that can help mitigate the impact of dyscalculia on a child’s academic progress.

Tailored Educational Support: Understanding a child’s specific difficulties can inform personalized teaching strategies and accommodations.

Emotional and Social Well-being: Addressing dyscalculia early can prevent associated issues such as low self-esteem and math anxiety, which can affect overall well-being.

## 12. Discussion on Key Discoveries and Study Outcomes

### 12.1. Limited Datasets

Obtaining public databases for dyscalculia research is challenging due to difficulties in capturing data from children with this specific NDD, often due to issues with concentration and focus. Additionally, there is a limited amount of data that specifically addresses the severity of dyscalculia. Given the complex nature of dyscalculia and its potential comorbidities, creating personalized AI tools for affected students presents significant challenges.

### 12.2. Ethics-Related Factors

Using AI-driven technologies to create individualized dyscalculic learning aids raises ethical questions. As a result of the widespread use of educational assistance technology with adolescents, issues related to informed consent, data security, and privacy become critical [56]. For example, student data should only contain information that is necessary for the intended use, such as building machine learning models [18]. Only after confirming that the person is informed and has given consent may data collection begin [19]. In order to protect students’ privacy and data, educators must also make sure that pupils understand the repercussions of utilizing such technologies [20]. Teachers could discuss the advantages and disadvantages of the technology without affecting the decision-making process in order to obtain approval [21]. Additionally, educational IT businesses have to abide by relevant laws. For instance, in the United States, the Children’s Online Privacy Protection Act [21] requires parental approval before collecting data on pupils younger than 13 years old. As a result, it is critical to put privacy first and make sure student data used to train machine learning models comply with regional ethical standards. When using assistive tools for dyscalculia, protective measures should be put in place to prevent data breaches, and ethical guidelines should be created before beginning any data gathering [61,62,63].

### 12.3. Limited Studies Related to AI in Dyscalculia

In all the studies reviewed, the majority focused on dyslexia, a learning disability related to reading difficulties. Only few studies specifically addressed dyscalculia, a math-related learning disability [64]. The remaining studies covered learning disabilities broadly. Half of the studies centered on school-age children, while others targeted individuals over 18, including university students, or did not specify age groups.

### 12.4. Research Questionnaire on the Role of AI in Supporting Dyscalculia

This study aimed to thoroughly investigate how AI has been utilized to support individuals with dyscalculia and other learning disabilities (LDs), going beyond its typical role in diagnosis. It focused on identifying practical ways in which teachers or students could employ AI to deliver personalized assistance to students already identified as having dyscalculia. The study aimed to answer these questions:

RQ:1 How to use artificial intelligence in the screening and therapeutics of dyscalculia?

Screening and therapeutics of dyscalculia are executed by incorporating artificial intelligence (AI) which includes reasoning techniques and knowledge representation. More notably, it uses such techniques as knowledge representation and reasoning (KRR), artificial neural networks (ANN), and case-based reasoning (CBR) to generate decision-making models that can help in screening and developing a treatment plan for dyscalculia.

These AI techniques are integrated into a cognitive approach that involves the following components:

1. Creating a structured understanding of dyscalculia in children, helping to pinpoint their challenges and assess the effectiveness of potential treatments.
2. Designing disMAT [60], a fun and educational mobile game tailored for children aged 5–10 who struggle with math, including dyscalculia, to serve both as a therapeutic tool and a screening method.
3. Implementing a cognitive engine, a system equipped with reasoning algorithms, to analyze data from the disMAT game and Discalculia Web app. This engine offers screening insights and recommends therapeutic activities, which are then validated by experts.
4. Designing tasks within the disMAT game to address specific difficulties faced by children with dyscalculia, such as memory, attention, and processing speed. These tasks are crafted to be engaging, encouraging children to participate willingly.

This is to ensure that the artificial intelligence system is open and develops according to the new technologies and the feedback resulting from it to serve as an accurate and efficient solution to diagnose and treat dyscalculia effectively.

RQ:2 What are the goals and challenges in creating a cognitive approach to dyscalculia using current technologies?

The goals and challenges in creating a cognitive approach to dyscalculia using current technologies are multifaceted and involve several key aspects:

1. Formal Knowledge Representation: The research aims to provide a formal knowledge account of dyscalculia in children, which includes identifying their difficulties and analyzing the outcomes of appropriate therapies.
2. Intelligent and Cognitive Proposal: The research seeks to develop an intelligent and cognitive proposal for the dyscalculia status in Portuguese children under logical formalisms.
3. Innovative and Dynamic Solution: The goal is to design and develop an innovative and dynamic solution for the therapy and diagnosis of dyscalculia in the form of a mobile game, which is engaging and educational for children.
4. Deductive–Inductive Methodology: The adopted methodology is deductive–inductive, which involves conceptual theory analysis and practical solutions.
5. Cognitive Engine: The creation of a cognitive engine that hosts reasoning algorithms such as knowledge representation and reasoning (KRR), artificial neural networks (ANN), and case-based reasoning (CBR) to provide inferences from the analysis of results.
6. Game-Based Solution: The development of a game-based solution, which is a mobile application that runs on the Android operating system, the most common in the target population.
7. Open-Source and Free Availability: The software and game-based solution are intended to be open-source and freely available for children, educators, and the general public.
8. Integration with Brain–Computer Interface (BCI): Exploring the integration of a BCI system to train the neuroplasticity of the brain in children with dyscalculia, potentially providing a tool for visual training.
9. Multidisciplinary Approach: Recognizing the multidisciplinary nature of dyscalculia and establishing collaborations with various departments and institutes to incorporate knowledge from different areas.
10. Ethical and Cost Considerations: Addressing the moral and ethical implications, as well as the cost of using non-invasive exams like EEG, fMRI, and MRI for more accurate information on dyscalculia [63].
11. Comorbidities and Learning Impairments: Studying the connections between dyscalculia and other comorbidities to improve screening and therapeutics.
12. Healthcare and AI: Applying artificial intelligence resources to improve healthcare processes, leading to quicker diagnoses and adequate therapeutic plans.

The challenges include the need for extensive research to develop effective tasks that address the fragile areas affected by dyscalculia, the creation of appealing and efficient therapies for children, and the establishment of partnerships to enhance the understanding and treatment of dyscalculia.

RQ:3 In the past 15 years, what AI-based solutions have emerged to aid students facing learning disabilities?

In the last 15 years, several AI applications have been developed to support students with learning disabilities (SWLDs). These applications are designed to provide individualized support and adapt to the diverse needs of SWLDs. The seven types of AI applications identified in the literature are as follows:

1. Adaptive learning: AI systems that customize learning materials based on individual student needs, often through intelligent tutoring systems or serious learning games.
2. Facial expression recognition: technology that analyzes students’ facial expressions to predict engagement levels, helping teachers understand which activities are most engaging for SWLDs.
3. Chat robots (chatbots): AI-driven tools that interact with students via text or voice to provide feedback, answer questions, and offer accessibility resources.
4. Communication assistants: AI applications that help SWLDs with written communication, such as by reading aloud or assisting with text composition.
5. Mastery learning: AI systems that track student progress and support them in achieving mastery of a skill through repeated practice and evaluation.
6. Intelligent tutors: AI-based tutoring systems that use machine learning to identify individual learning difficulties and recommend personalized learning strategies.
7. Interactive robots: robots that use AI to physically interact with students and predict their engagement in the classroom using multimodal learning.

These AI applications have been integrated into educational settings to support SWLDs in various ways, from providing personalized learning experiences to enhancing social and emotional growth. The review also noted that adaptive learning was the most widely used type of AI technology among the studies examined.

## 13. Conclusions

This review delved into the potential of AI in assisting individuals with specific learning disabilities, with an emphasis on dyscalculia. Most studies predominantly focused on dyslexia, a learning disability associated with reading challenges, while only a few specifically addressed dyscalculia, a math-related learning disability. The remaining studies took a broader approach to learning disabilities. Interestingly, half of the studies targeted school-age children, highlighting the importance of early intervention, while others focused on individuals over 18, including university students, or did not specify age groups. Future research should concentrate on assessing the usability and effectiveness of AI tools tailored for dyscalculia to enhance their educational experiences. In conclusion, learning disabilities such as dyslexia, dysgraphia, and dyscalculia significantly impact an individual’s cognitive abilities, presenting challenges in reading, writing, and mathematical comprehension. These disorders, categorized under neurodevelopmental disorders (NDDs), affect 10–20% of children globally, with comorbidities often complicating their management. Dyscalculia, specifically, affects approximately 6% of individuals with learning disabilities and can persist into adulthood without early intervention. Despite the availability of specialized tests for diagnosis, the process remains manual and time-consuming. Technological advancements in the IoT, AI, and ML offer promising solutions to enhance dyscalculia detection and intervention, with machine learning algorithms showing potential in automating screening processes. However, further research is needed to address challenges such as obtaining comprehensive datasets, ensuring data privacy and consent, and expanding studies specifically focused on dyscalculia. Collaborative efforts combining behavioral studies, neuroimaging, and cognitive assessments are essential to advance understanding, develop effective interventions, and improve outcomes for individuals with dyscalculia and other learning disabilities.

## Figures and Tables

**Table 1:** Overview of Technological Interventions for Learning Disabilities.

Year	Technology Used	Prototype	Methodology	Effectiveness	Disability Focused
2013 [44]	AI technology	Bayesian network.	Using AI technology, the material was modified to fit the user’s requirements or learning style.	Increases the learning rate.	Dyscalculia
2019 [45,46]	Android application	Digital tool.	Developing digital tools.	Improving instruction by having students practice words, numerals, and alphabets while taking into account their choice for digital resources.	Dyslexia
2019 [47]	Adaptive educational hypermedia system employing AI to deliver personalized online learning interventions	Novel adaptive e-learning system named Dyslexia Adaptive E-Learning Management System (DAELMS) designed to cater to students’ learning styles and dyslexia types.	Implementation and adaptation algorithms.	Enhanced learning outcomes when the system matched learning materials to the user’s dyslexia type or learning style.	Dyslexia
2020 [49]	Intelligent serious games	Intelligent serious games tailored to accessible learning objectives, targeting the enhancement of social and cognitive competences in children with learning disabilities.	A 4-step methodology was employed: needs analysis, content development, intelligent serious game creation, and usability evaluation based on teacher and student feedback.	Significant improvements in social and cognitive competences after using the intelligent serious games. Pilot tests across participating countries further validated these findings.	Learning disabilities
2021 [48]	AI and virtual reality	BESPECIAL utilizes clinical reports, self-assessment questionnaires, and psychometric test results to extract information about dyslexic students’ needs. It employs AI for predicting suitable support methodologies and virtual reality for engagement and data collection.	Two-stage implementation: firstly, training the AI with extensive data to create a category-specific predictor, followed by student-specific adjustments based on evaluations and feedback from users.	Preliminary results from approximately 700 dyslexic students identified concentration, memory impairments, and scheduling as primary challenges. Effective support strategies included clear material layout, use of images, and taking pauses during lessons, emphasizing human interaction over machine-based solutions.	Dyslexic
2018 [56]	Advanced ML techniques	Dyslexia Adaptive Learning Model designed to predict student engagement levels by analyzing facial images.	Frontal face image analysis and classification algorithm used.	High accuracy rates ranging from 97% to 97.8%.	Dyslexia
2022 [29,30]	Augmented reality	Gaming.	Design thinking methodology.	To improve children’s comprehension skills.	Dyscalculia
2022 [39]		Assistive technology tool.	Computer aided instruction.	To enhance dyscalculic children’s learning of mathematics from the pre-test to the post-test.	Dyscalculia
